# Uncoded chronic kidney disease prevalence in secondary care: a retrospective audit with population health implications

**DOI:** 10.1186/s12882-025-03967-x

**Published:** 2025-01-24

**Authors:** Samantha Dolan, Ajitesh Anand, Philip A. Kalra, Stuart Stewart

**Affiliations:** 1 Rochdale Care Organisation, Northern Care Alliance NHS Foundation Trust, Rochdale, England; 2 Manchester Medical School, The University of Manchester, Oxford Road, Manchester, England; 3 Donal O’Donoghue Renal Research Centre, Northern Care Alliance NHS Foundation Trust, Salford, England; 4 Centre for Primary Care & Health Services Research, The University of Manchester, Manchester, England

**Keywords:** Chronic kidney disease, Primary health care, Diagnosis, Coding, Uncoded

## Abstract

**Background:**

One million patients are estimated to have undiagnosed chronic kidney disease (CKD) in England. Clinical coding in CKD is associated with improved management and lower acute kidney injury (AKI), unscheduled care and mortality risk. Primary care’s role in coding CKD is well documented. However, there is scant evidence on CKD coding quality in secondary care. Primary aims: to measure total and coded/uncoded CKD prevalence on admission and discharge, and conversion of uncoded to coded CKD in secondary care. Secondary aims: to map coding status to kidney health inequality themes and to measure predictors of coding, death and AKI.

**Methods:**

Retrospective audit in an acute medical hospital ward in England, April 2022-February 2023. Descriptive statistics include counts/percentages for categorical data, prevalence estimates and rates. Logistic regression measured significant predictors (p = < 0.05) of receiving a diagnostic CKD code on discharge, risk of death, and of AKI.

**Results:**

Uncoded CKD prevalence using discharge estimated GFR (eGFR) was 58.7% (*n* = 283), equating to 1.1 cases uncoded CKD per bed/month and 13.7 cases uncoded CKD per bed/year. Conversion of uncoded to coded CKD at discharge was only 6.7%. Hypertension and advanced CKD were significant predictors of coding CKD on discharge in uncoded patients. Age, sex, indices of multiple deprivation, and AKI were significant predictors of death during admission. Advanced CKD was a significant predictor of AKI during admission.

**Conclusions:**

Uncoded CKD is highly prevalent in an acute medical hospital ward highlighting opportunity to improve coding in another part of the health system in addition primary care.

**Supplementary Information:**

The online version contains supplementary material available at 10.1186/s12882-025-03967-x.

## Introduction

Primary care’s role in detecting and diagnosing chronic kidney disease (CKD) is well documented [1–3]. A recent review highlighting extensive barriers and enablers to effective CKD care in UK primary care [2] sheds light on an often overlooked but major finding from the national CKD audit (NCKDA); assigning a diagnostic clinical code for patients with CKD is associated with lower mortality risk, a decreased risk of acute kidney injury (AKI), and reduced risk of unplanned hospital admissions [4, 5]. In modern healthcare systems, clinical coding– the process of translating medical information into standardised codes– is essential for a range of clinical, administrative, financial and research purposes [6]. Until 2015 in England, general practitioners (GPs) were financially incentivised through the Quality and Outcomes Framework (QOF) to diagnose and clinically code patients with CKD [2]. Post-2015, GPs are still encouraged to maintain a register of patients with CKD by coding their diagnosis [7]. Specifically, clinical coding allows for easy identification of patients with CKD for routine chronic disease reviews, automated monitoring alerts, and vaccination priority [2, 8]. As such, quality improvement (QI) work in primary care has focused on increasing detection and diagnosis of undiagnosed and uncoded CKD [8]. However, there is scant evidence of the prevalence of uncoded CKD in a hospital setting and how effective secondary care clinicians are at coding CKD in hospitalised patients; such evidence could provide further avenues to diagnose and code the estimated one million people living with undiagnosed CKD in the England [9]. Furthermore, several kidney health inequality themes have been described however these have never been mapped across coding status [10, 11]. Doing so would allow greater understanding of how clinical coding interacts with health inequality themes to inform patient and population level interventions and policy to reduce kidney health inequalities.

The primary aims of this audit were to measure, the prevalence of CKD in patients admitted in a hospital general medical ward over a ~ 12-month period; the prevalence of coded/uncoded CKD; the conversion of uncoded to coded CKD in secondary care. Secondary aims were to map coding status to kidney health inequality themes [10] and to measure predictors of coding, death and AKI.

## Methodology

### Study design

Retrospective audit of inpatient admission data from 2nd April 2022 to 22nd February 2023 as part of a Population Health Fellowship in Chronic Kidney Disease and University of Manchester Medical School– medical student Applied Personal Excellence Project. Primary aims: to measure total and coded/uncoded CKD prevalence on admission and discharge, and conversion of uncoded to coded CKD in secondary care. Secondary aims: to map coding status to kidney health inequality themes and to measure predictors of coding, death and AKI. Diagnosis and coding of CKD are key standards within NICE CKD guidelines [1] and the primary care Quality and Outcomes Framework (QOF) [7], and are further supported by expert consensus [12].

### Setting, participants and data sources

All adult patients admitted to the 21-bed Clinical Assessment Unit in Rochdale Care Organisation (RCO), including multiple admissions by the same patients, were included. Inpatient admission data were extracted from the electronic health record (EHR)– HealthViews.

### Ethical approval

Ethical approval was not required for this audit as per UK Health Research Authority (HRA) guidance which was registered with and endorsed by the Northern Care Alliance (NCA) National Health Service (NHS) Foundation Trust Research and Innovation department (project code: 23HIP17).

### Variables

The following outcomes were used: prevalence estimates, calculated as the proportion of patients with the outcome of interest (numerator) divided by the total number of patients in the relevant group (denominator), expressed as a percentage; coded CKD based on admission or discharge eGFR and latest uACR in preceding 12 months; conversion from uncoded to coded CKD at discharge; odds of patients receiving a diagnostic CKD code on discharge, death during admission, and AKI during admission (defined according to KDIGO definition [13]).

Demographic and predictor variables included: age and age groups (17–29, 30–39, 40–49, 50–59, 60 − 59, 70–79, 80–89, 90 + years), sex, ethnicity, GP practice post code– mapped to indices of multiple deprivation (IMD) [14]: a measure of geographical area level deprivation at a low geographical level of approximately 1600 people, measured over several domains (income; employment; education, skills, and training; health deprivation and disability; crime; and housing) - used as a proxy for socioeconomic status; admission and discharge date; first and last creatinine on admission (µmol); uACR (mg/mmol); past diagnostic codes in primary care: CKD, diabetes, hypertension, mental health diagnoses (anxiety, depression, bipolar disorder, schizophrenia and psychosis, self-harm and suicidal ideation, eating disorder, dementia), cancer; diagnoses of AKI and/or heart failure during admission; death during admission. Derived variables included eGFR (ml/min/1.73m^2^) using the CKD-EPI 2021 equation [15]. CKD was defined and staged according to the KDIGO classification criteria using 2 x eGFR values within 90 days and the latest uACR in the preceding 12 months (if measured) [3]; patients with eGFR > 60 ml/min/1.73m^2^ without uACR were classified as ‘Not CKD’. Admission eGFR was the first eGFR during admission. Discharge eGFR was the last eGFR during admission. For patients with only one eGFR measured on admission, this was carried forwards as their discharge eGFR. Coded CKD was defined as any patient with biochemical evidence of CKD who either had a diagnostic CKD code in their primary care EHR or who were given a diagnostic CKD code on discharge from hospital. Patients with coded CKD were accepted as having CKD and this diagnosis was not challenged. Uncoded CKD was defined as any patient with biochemical evidence of CKD on discharge without ever having a diagnostic CKD code in their primary care EHR. Kidney health inequality themes are overarching themes which highlight patterns of disparities in kidney health outcomes for patients. Coding status was mapped to kidney health inequality themes of sex, age, ethnicity, socioeconomic status, and mental health diagnoses [10].

### Bias

To minimise selection and information bias we included all admitted patients within the prespecified study period and cross-checked hospital level data with primary care EHR data to accurately identify patients with uncoded and coded CKD.

### Statistical analyses

R and R Studio were used for data cleaning and analysis, employing packages dplyr, tidyr, logistf, patchwork and ggplot2. Descriptive statistics (counts/percentages for categorical data; mean and standard deviation for continuous data) were reported. Demographics were compared between the total cohort and patients with CKD. Firth’s penalised likelihood method was used for logistic regression analyses to address the sample size limitations and potential separation [16, 17]. Analyses assessed key predictors of diagnostic coding at discharge, in-hospital mortality, and AKI risk during admission. An alpha level of 0.05 was set a priori and 95% confidence intervals (CIs) are presented. Logistic regression assumptions were verified before modelling. Likelihood ratio tests and p-values guided model selection.

## Results

Overall, 1364 patients were admitted over 1520 admissions; CKD prevalence was 35.3% with 482 patients admitted over 550 admissions (Table 1). Of 1364 patients, 53.4% were female (*n* = 728); of 482 patients with CKD, 45.9% (*n* = 221) were female. White British ethnicity was the most prevalent (81.4% and 83.0%, total and CKD population, respectively), followed by Asian Pakistani (11.3% and 8.7%), and White Irish (1.7% and 2.7%). The most frequently observed age group was 80–89 years (292 patients; 21.4% prevalence and 163 patients; 33.8% prevalence, respectively). Approximately two-thirds of patients were living in the 3 most deprived deciles.

Specifically for patients with CKD, 26.7% (*n* = 129) had one eGFR measured, and 71.8% (*n* = 346) had two or more measured; uACR testing prevalence in the CKD cohort was 58.3%. Of those tested, microalbuminuria (uACR 3–30 mg/mmol) prevalence was 35.7% and macroalbuminuria (uACR > 30 mg/mmol) prevalence was 14.3%. Diabetes prevalence was 34.9%; hypertension prevalence was 58.1%; any MH diagnosis prevalence was 15.6%; depression prevalence was 11.6%; dementia prevalence was 7.5%; history of cancer prevalence was 18.9%. AKI incidence during admission was 29.6%; acute heart failure incidence during admission was 20.5%.

Inpatient mortality rate was 7.5% for patients with coded CKD in primary care versus 4.8% for patients without CKD in primary care. Given there were 283 cases of uncoded CKD in the 21-bed medical unit, there were 1.1 cases of uncoded CKD per bed/month, and 13.7 cases of uncoded CKD per bed/year (Table 2).

**Table 1:** Demographics and clinical characteristics summary table for entire cohort and CKD cohort

	Entire cohort	CKD^a^ only		Entire cohort	CKD^a^ only
	*N* (%)	*N* (%)		*N* (%)	*N* (%)
**Total patients**	1364	482 (35.3)	**Total patients**	1364	482 (35.3)
**Total admissions**	1520	550 (36.2)	**Total admissions**	1520	550 (36.2)
**Variables**					
**Sex ** ^b^			**eGFR testing ** ^b^		
Male	636 (46.6)	261 (54.1)	0	64 (4.7)	7 (1.5)
**Ethnicity** ^b^			1	466 (34.2)	129 (26.7)
White British	1096 (81.4)	400 (83.0)	2 or more	834 (61.1)	346 (71.8)
White Irish	23 (1.7)	13 (2.7)	**ACR testing** ^b^		
White Other	16 (1.2)	5 (1.0)	uACR measured	420 (30.8)	281 (58.3)
Asian Pakistani	152 (11.3)	42 (8.7)	**Previous diagnoses ** ^b^		
Asian Bangladeshi	18 (1.3)	6 (1.2)	Diabetes	293 (21.5)	168 (34.9)
Asian Indian	3 (0.2)	2 (0.4)	Hypertension	531 (38.9)	280 (58.1)
Asian Chinese	1 (0.1)	0 (0.0)	Any MH diagnosis	272 (19.9)	75 (15.6)
Black African	15 (1.1)	4 (0.8)	Anxiety only	127 (9.3)	32 (6.6)
Black Other	4 (0.3)	2 (0.4)	Depression only	183 (13.4)	56 (11.6)
Other ethnic groups	18 (1.3)	5 (1.0)	Anxiety with Depression	87 (6.4)	25 (5.2)
Missing	18 (1.3)	3 (0.5)	Eating disorder	2 (0.1)	0 (0.0)
**Age** ^b^			Self-harm	7 (0.5)	0 (0.0)
Age mean (SD)	65.7 (19.7)	75.7 (14.5)	Schizophrenia	22 (1.6)	4 (0.8)
**Age groups** ^b^			Bipolar disorder	10 (0.7)	4 (0.8)
17–29	95 (7.0)	5 (1.0)	Dementia	64 (4.7)	36 (7.5)
30–39	94 (6.9)	8 (1.7)	Cancer	204 (15.0)	91 (18.9)
40–49	108 (7.9)	17 (3.5)	**Diagnoses during admission ** ^c^		
50–59	187 (13.7)	35 (7.3)	Acute kidney injury	226 (14.9)	163 (29.6)
60–69	213 (15.6)	62 (12.9)	Heart failure	181 (11.9)	113 (20.5)
70–79	280 (20.5)	130 (27.0)			
80–89	292 (21.4)	163 (33.8)			
90+	95 (7.0)	62 (12.9)			
**IMD** ^b^					
1 (most deprived)	636 (48.6)	235 (48.8)			
2	60 (4.6)	15 (3.1)			
3	214 (16.4)	69 (14.3)			
4	41 (3.1)	15 (3.1)			
5	125 (9.6)	80 (16.6)			
6	125 (9.6)	45 (9.3)			
7	4 (0.3)	2 (0.4)			
8	9 (0.7)	5 (1.0)			
9	6 (0.5)	1 (0.2)			
10 (least deprived)	0 (0.0)	0 (0.0)			
Missing	56 (4.1)	15 (3.1)			

Key: ^a^ biochemical evidence of CKD using discharge eGFR and uACR in the last 12 months AND patients with coded CKD on admission; ^b^ based on total patients not total admissions; ^c^ based on total admissions not total patients

**Table 2:** Coded and uncoded CKD summary from admission to discharge; and cases of uncoded CKD per bed/month and per bed/year

	Entire cohort	Total CKD based on coded on admission and CKD on discharge	Coded on discharge by secondary care of 283 patients with uncoded CKD (excluding died during admission)	Remained uncoded on discharge (excluding died during admission)		
**CKD status**	CKD and not CKD	Coded and Uncoded CKD	Coded (in secondary care) CKD	Uncoded (in secondary care) CKD	Cases of uncoded CKD per bed/month	Cases of uncoded CKD per bed/year
**Total**	1364	482*	19	264	1.1	13.7

Key: * 199 Patients with coded CKD in primary care on admission + 283 Patients with CKD based on discharge eGFR (and uACR in last 12 months) that were uncoded on admission (excluding died during admission) = 482; Cases per bed/month = 264 cases / (21 beds * 11 months) = 1.14 cases per bed/month; Cases per bed/year = 1.14 * 12 = 13.7 cases per bed/year

### Prevalence of coded and uncoded CKD

**Fig. 1:**
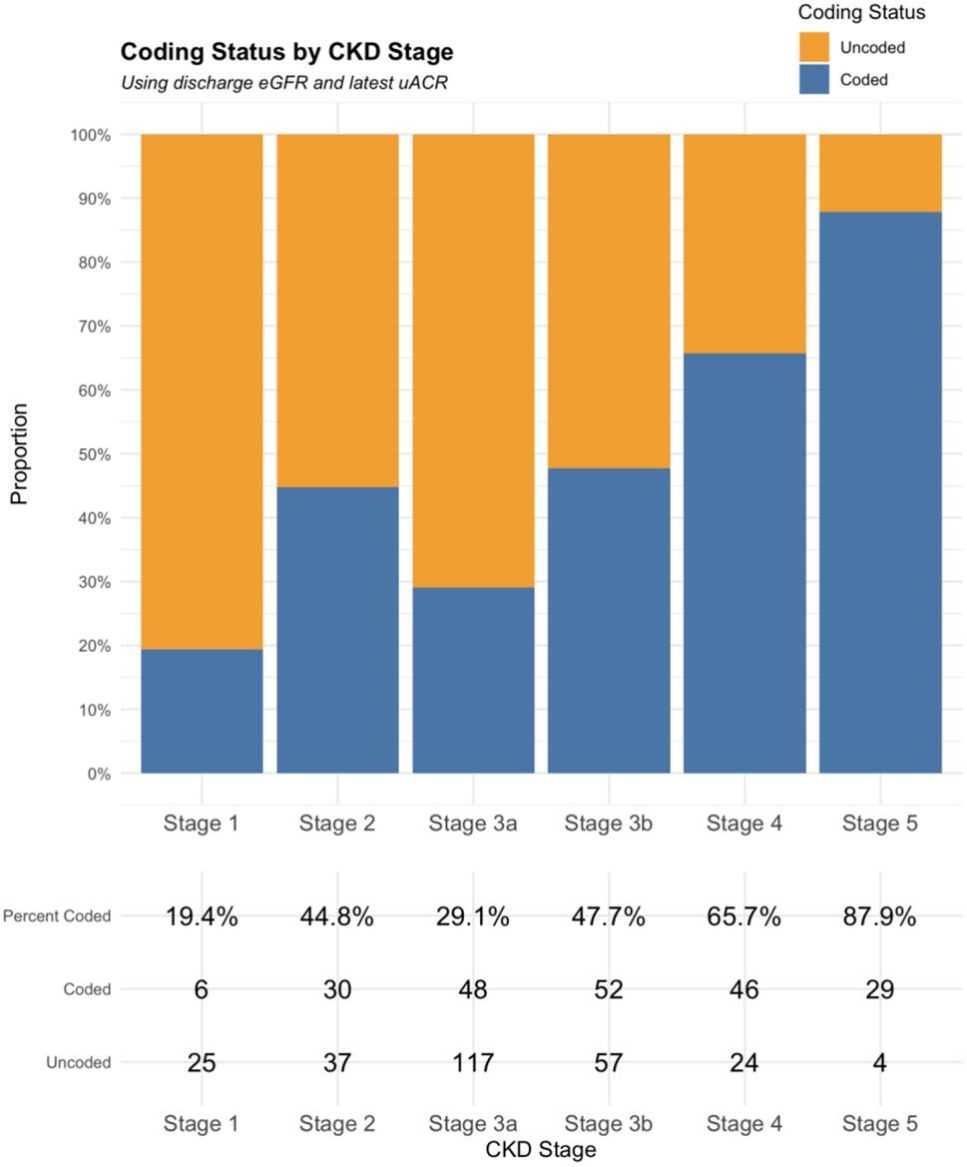
Coding status by CKD stage– using discharge eGFR to calculate CKD stages. (7 cases with no eGFR measurements excluded)

Of 482 patients with CKD, 199 (41.3%) were coded in primary care on admission and a further 283 (58.7%) had evidence of CKD using discharge eGFR and uACR but were uncoded in primary care. Of patients with uncoded CKD (*n* = 283), only 19 were coded on discharge, representing a 6.7% conversion of uncoded to coded CKD, leaving 264 patients with uncoded CKD. Figure 1 shows the prevalence of uncoded CKD generally decreases as CKD stage increases. Uncoded CKD was more common up until stage G4.

### Mapping coding status to kidney health inequality themes

**Fig. 2:**
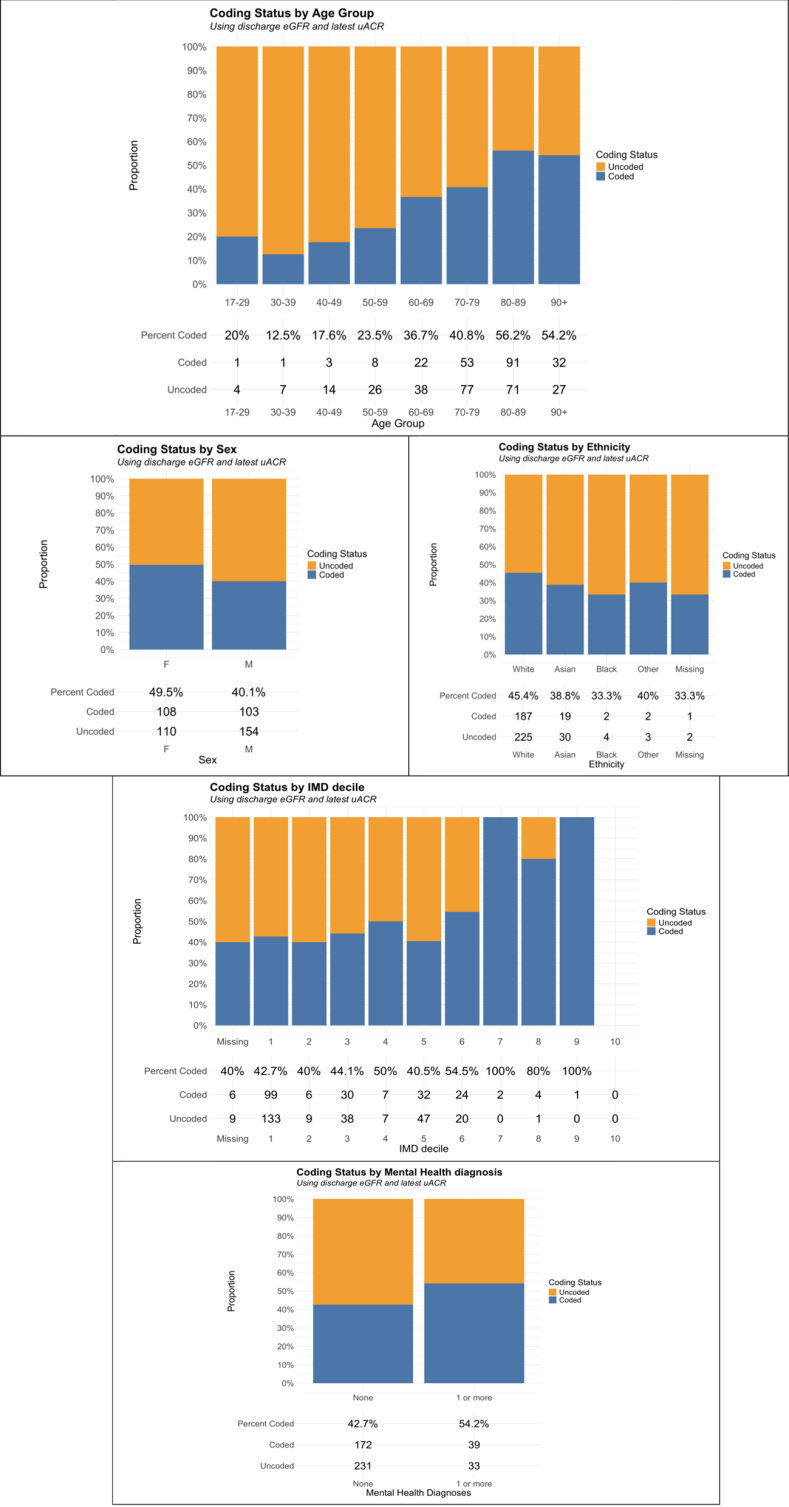
Graphical summary of coding status mapped to 5 health inequality themes [10]

Figure 2 highlights coding status across 5 kidney health inequality themes. Age: coded CKD was more common as age increases. Sex: Less than half of males or females had coded CKD. Ethnicity: Less than half of all ethnicities had coded CKD. Socioeconomic status: in general, coded CKD becomes more common as deprivation decreases. Mental health: coded CKD is more common in patients with 1 or more MH diagnoes. Overall, across health inequality themes, uncoded CKD was generally more common than coded CKD.

### Diabetes prevalence and CKD coding status

**Fig. 3:**
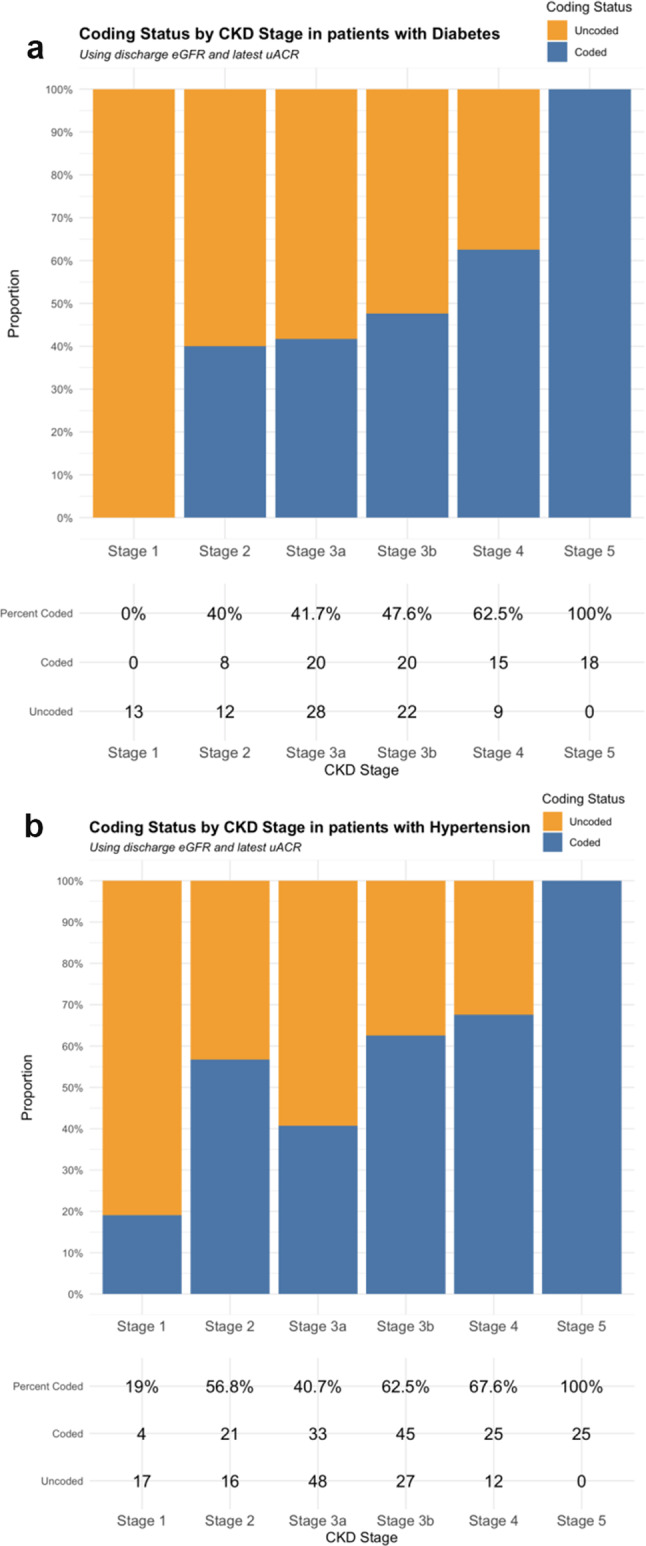
(**a**): Coding status by CKD stage in patients with diabetes. Total = 165 (not 168) as 3 patents did not have eGFR measured during admission. (**b**): Coding status by CKD stage in patients with hypertension. Total = 273 (not 280) as 7 patents did not have eGFR measured during admission.

Of 482 patients with CKD, 168 (34.9%) had diabetes; uACR was measured in 123 (73.2%). Figure 3a shows there were no patients with diabetes and coded CKD stage 1, and uncoded CKD was more common until stage 4. There were no patients with diabetes and uncoded CKD stage 5.

### Hypertension prevalence and CKD coding status

Of 482 patients with CKD, 280 (58.1%) had hypertension; uACR was measured in 173 (61.8%). Figure 3b shows coded CKD generally becomes more common as CKD stage increases. There were no patients with hypertension and uncoded CKD stage 5.

### Predictors of outcomes

#### Predictors of coding on discharge: uncoded CKD

Multivariable Firth’s logistic regression (Table 3: final model) showed in patients with uncoded CKD, hypertension (OR 3.14; CIs 1.14–9.97) and advanced CKD (OR 4.90; CIs 1.60–14.20) were significant predictors of receiving a diagnostic CKD code on discharge.

**Table 3:** Multivariable Firth's logistic regression model of predictors of CKD coding on discharge in patients with uncoded CKD (discharge eGFR and uACR)

	Initial model				Final model			
	Estimate	SE	*p*-value	95% CIs	Estimate	SE	*p*-value	95% CIs
**Age**								
Age	1.04	1.02	0.07	1.00 to 1.09	1.04	1.02	0.08	1.00 to 1.08
**Sex**								
Male	0.75	1.63	0.60	0.25 to 2.18	-	-	-	-
**Ethnicity**								
Non-White	0.60	2.19	0.54	0.09 to 2.90	-	-	-	-
**Socioeconomic deprivation**								
IMD	1.09	1.12	0.50	0.85 to 1.40	-	-	-	-
**Diagnoses**								
Diabetes	1.64	1.71	0.40	0.51 to 5.32	-	-	-	-
Hypertension	2.77	1.67	0.06	0.95 to 9.09	3.14	1.70	**0.03**	1.14 to 9.97
Dementia	1.08	2.41	0.94	0.11 to 5.60	-	-	-	-
MH diagnosis	1.19	1.94	0.82	0.22 to 4.35	-	-	-	-
Cancer diagnosis	1.28	1.79	0.70	0.34 to 4.16	-	-	-	-
AKI during admission	1.89	1.61	0.23	0.65 to 5.16	-	-	-	-
HF during admission	0.66	1.86	0.52	0.15 to 2.24	-	-	-	-
**Urine ACR**								
ACR > 30 mg/mmol	3.44	2.53	0.22	0.47 to 28.88	-	-	-	-
ACR 3–30 mg/mmol	1.22	2.14	0.81	0.26 to 7.75	-	-	-	-
**CKD Stage**								
Advanced CKD (stages 4 and 5 combined)	3.67	1.72	**0.03**	1.14 to 11.51	4.90	1.71	**0.006**	1.60 to 14.20
**Table Key**								
Reference categories	Sex: Female; Ethnicity: White; Diagnoses: absence of diagnosis; ACR: ACR < 3mg/mmol; CKD stage: stages 1, 2 and 3 combined. SE = standard error							
Statistically significant	values in **bold.**							

### Predictors of death during admission

In patients with CKD, age (OR 1.07; CIs 1.01–1.16), sex (OR 0.27; CIs 0.05-1.00) IMD decile (OR 0.62; CIs 0.32–0.94) and AKI during admission (OR 3.92; CIs 1.14–14.82) were all significant predictors of death during admission (supplement 8.1: final model).

### Predictors of AKI during admission

In patients with CKD, advanced CKD (OR 3.08; CIs 1.96–4.88) was the only significant predictor of AKI during admission (supplement 8.2: final model).

## Discussion

Overall CKD prevalence was 35.3%. Uncoded CKD prevalence at discharge was 58.7%. Conversion of uncoded CKD on admission to coded CKD at discharge was only 6.7%. Uncoded CKD was generally more common across kidney health inequality themes. Hypertension and advanced CKD stages were significant predictors of coding on discharge in those patients with uncoded CKD at discharge. Age, IMD deciles and AKI during admission were significant predictors of death during admission; and advanced CKD stages were significant predictors of AKI during admission. Our key finding is the low conversion rate from uncoded to coded CKD of just 6.7% which hasn’t been shown before in hospital settings but has implications for improving clinical practice and population health.

Our findings have several implications. First, whilst we show how the prevalence of uncoded CKD is different in hospital, we also demonstrate the universality of the problem of uncoded CKD that crosses traditional boundaries of healthcare, necessitating a collaborative and population health approach. Future QI work at RCO seeks to automate coding and diagnosis of patients with biochemical evidence of CKD on discharge to trigger a primary care chronic disease review with subsequent re-audit. Second, a low conversion rate of uncoded to coded CKD reflects vast missed opportunities to diagnose CKD and reduce future health risks, but also sets the benchmark for further hospital-based QI work and research. Third, our data reveals approximately 1.1 cases of uncoded CKD per bed/month, and 13.7 cases of uncoded CKD per bed/year– useful metrics for visualising the frequency in which healthcare professionals come into contact with uncoded CKD in an acute medical ward in a hospital setting and for measuring the efficacy of future interventions. In a hospital with 400 acute medical beds, this would equate to 5472 cases of uncoded CKD per year. Fourth, a general lack of significant predictors of receiving a diagnostic CKD code on discharge highlights the somewhat random nature of clinical coding behaviour in this setting necessitating further exploratory qualitative work and clinical education that has shown to be effective in primary care [8, 18]. Finally, EHRs and clinical laboratory reporting systems in secondary care are capable of alerting clinicians when a patient has uncoded CKD, and doing so at scale– a key tool of population health strategies. Such an opportunity for intervention is low hanging fruit for addressing the estimated one million undiagnosed cases of CKD in England [9]. As seen in primary care [8, 19], QI work and research must urgently address the major burden of undiagnosed CKD from the newly described vantage point of secondary care.

### Comparison with existing literature

There is scant evidence of measurement of CKD prevalence in hospitalised patients. Our study demonstrates a greater prevalence of CKD in acute medical inpatients (35.3%) than existing observational research: 14.8% of 13,383 adult inpatients in China [20]; and 12.7% in 826 adult acute medical inpatients in Brazil [21] which may be a function of our study population being older, living in greater levels of deprivation, with different access to healthcare, and in an acute medical ward. Such a finding aligns with kidney health inequality data showing that older adults not only face barriers to diagnosis but also effective CKD care [11]. Nevertheless, earlier stages of CKD are coded less often compared to later stages of CKD, a pattern observed in existing research [2, 4]. This discrepancy arises from factors including asymptomatic earlier stages of CKD, variable clinical recognition, limited screening, and competing demands in primary care [2, 8, 22]. Existing evidence focuses on primary care as a central setting for improved detection, diagnosis and coding of CKD [2]; however, our findings highlight not only the high prevalence of uncoded CKD in a hospital setting but therefore the opportunity for intervention in an additional setting to primary care. This aligns with the national strategy for CKD care highlighting the role of all healthcare professionals in addressing kidney health inequalities [11]. Furthermore, coding a diagnosis of CKD increases the chances of optimal management and better health outcomes [4, 5]. We show that a diagnosis of hypertension (OR 3.14; CIs 1.14–9.97) and advanced CKD (OR 4.90; CIs 1.60–14.20) significantly increase the odds of patients with uncoded CKD receiving a diagnostic CKD code on discharge. Whilst these predictors have wide CIs indicating some uncertainty in the exact estimates, such predictors are clinically sound and correlate with existing evidence [2, 4]. Observational research also shows uncoded CKD in a hospitalised older adult population was 50.8% and associated with higher inappropriate prescribing of medications [23] emphasising the clinical implications of uncoded CKD in a hospital setting and for a vulnerable patient population. Furthermore, patients with uncoded CKD may be at greater risk of having renoprotective medication stopped and not restarted. In our study, uncoded CKD was generally more prevalent than coded CKD across kidney health inequality themes demonstrating the potential for coding as an intervention to have equity across themes. We show uACR testing is suboptimal and below guideline recommendations for high-risk patients (diabetes/hypertension) [1]. Our findings also contribute to the overall limited evidence base of mental health disease prevalence in patients with CKD [10, 24–27].

### Strengths and limitations

Undiagnosed and uncoded CKD is often considered a primary care problem. However, there is a notable gap in the evidence examining this issue within hospital settings. Our study addresses this gap by thoroughly auditing inpatient admissions and correlating these findings with primary care EHRs diagnoses. This approach allows for a comprehensive understanding of a patient’s health status, presenting a new view of the problem of uncoded CKD from another vantage point. Additionally, our study aligns findings relating to coding status with the latest evidence on kidney health inequalities to add further colour to this picture. To our knowledge we present the first evidence on conversion rates of uncoded to coded CKD in a hospital setting which sets the benchmark for future research. Limitations include that eGFR fluctuates during acute illness which may overestimate CKD stage. IMD was calculated on GP practice postcode, not patient postcode; GP practices were heavily based in areas of IMD deciles 1–3 where uncoded CKD prevalence is higher, potentially limiting generalisability to other populations. Missing uACR underestimates earlier stages of CKD and risk of poor outcomes. uACR was included from within the previous 12 months, not necessarily at the time of eGFR testing during the hospital admission. All analyses were of routinely collected data which underreport true CKD prevalence. Furthermore, the retrospective nature of our study carries a risk of residual confounding.

## Conclusions

Uncoded CKD was highly prevalent in patients admitted to this secondary care setting. Conversion of uncoded to coded CKD on discharge from hospital was low representing multiple missed opportunities for improving kidney care and health outcomes and reducing health inequalities.

## Electronic supplementary material

Below is the link to the electronic supplementary material.

Supplementary Material 1

## Data Availability

The datasets generated and/or analysed during the current study are not publicly available due to them being part of ongoing quality improvement activity. Additionally, the NCA NHS foundation trust do not make clinical audit data available to the public.

## Abbreviations

ACR: Albumin creatinine ratio
AKI: Acute kidney injury
CI/s: Confidence interval/s
CKD: Chronic Kidney Disease
CKD-EPI: Chronic Kidney Disease Epidemiology Collaboration
EHR: Electronic health record
GP: General Practitioner
IMD: Indices of Multiple Deprivation
KDIGO: Kidney Disease Improving Global Outcomes
MH: Mental health
NCA: Northern Care Alliance
NHS: National Health Service
OR: Odds ratio
QI: Quality improvement
uACR: Urine albumin creatinine ratio
