# Supplementary material for: Uncoded chronic kidney disease prevalence in secondary care: a retrospective audit with population health implications
Source: BMC Nephrol. 2025 Jan 24;26:39. doi: 10.1186/s12882-025-03967-x (PMC11762104; doi:10.1186/s12882-025-03967-x)
Supplement: Supplementary file 1 — Supplementary Material 1 [file 12882_2025_3967_MOESM1_ESM.docx]

Supplementary data

Multivariable Firth’s logistic regression model of predictors of death during admission

|  | **Initial Model** | | | | **Final Model** | | | |
| --- | --- | --- | --- | --- | --- | --- | --- | --- |
|  | **Estimate** | **SE** | **p-value** | **95% CIs** | **Estimate** | **SE** | **p-value** | **95% CIs** |
| **Age** | | | | | | | | |
| Age | 1.05 | 1.03 | 0.15 | 0.98 to 1.14 | 1.07 | 1.03 | **0.03** | 1.01 to 1.16 |
| **Sex** | | | | | | | | |
| Male | 0.27 | 1.85 | 0.07 | 0.05 to 1.08 | 0.27 | 2.00 | **0.05** | 0.05 to 1.00 |
| **Ethnicity** | | | | | | | | |
| Non-White | 0.43 | 2.33 | 0.37 | 0.04 to 2.50 | - | - | - | - |
| **Socioeconomic deprivation** | | | | | | | | |
| IMD | 0.69 | 1.21 | 0.08 | 0.36 to 1.05 | 0.62 | 1.26 | **0.02** | 0.32 to 0.94 |
| **Diagnoses** | | | | | | | | |
| Diabetes | 1.33 | 1.81 | 0.70 | 0.30 to 5.51 | - | - | - | - |
| Hypertension | 3.96 | 2.14 | 0.10 | 0.77 to 40.97 | - | - | **-** | - |
| Dementia | 0.18 | 3.60 | 0.18 | 0.00 to 1.88 | - | - | - | - |
| MH diagnosis | 1.72 | 1.94 | 0.52 | 0.28 to 7.86 | - | - | - | - |
| Cancer diagnosis | 0.94 | 1.96 | 0.95 | 0.14 to 4.22 | - | - | - | - |
| AKI during admission | 3.25 | 1.72 | 0.07 | 0.90 to 12.61 | 3.92 | 1.83 | **0.03** | 1.14 to 14.82 |
| HF during admission | 0.58 | 2.00 | 0.50 | 0.08 to 2.56 | - | - | - | - |
| **Urine ACR** | | | | | | | | |
| ACR > 30 mg/mmol | 1.39 | 3.06 | 0.81 | 0.08 to 23.81 | - | - | - | - |
| ACR 3–30 mg/mmol | 0.43 | 2.88 | 0.52 | 0.03 to 6.24 | - | - | - | - |
| **CKD Stage** | | | | | | | | |
| Advanced CKD (stages 4 and 5) | 2.14 | 1.91 | 0.35 | 0.41 to 10.36 | - | - | - | - |
| **Table Key** | | | | | | | | |
| *Reference categories* | Age: 1 unit year increase; Sex: Female; Ethnicity: White; Diagnoses: absence of diagnosis; Urine ACR: ACR < 3; CKD stage: stages 1, 2 and 3 combined. SE = standard error | | | | | | | |
| *Statistically significant* | values in **bold.** | | | | | | | |

- 1. Multivariable Firth’s logistic regression model of predictors of AKI during admission

|  | **Initial Model** | | | | **Final Model** | | | |
| --- | --- | --- | --- | --- | --- | --- | --- | --- |
|  | **Estimate** | **SE** | **p-value** | **95% CIs** | **Estimate** | **SE** | **p-value** | **95% CIs** |
| **Age** | | | | | | | | |
| Age | 0.99 | 1.01 | 0.21 | 0.97 to 1.01 | - | - | - | - |
| **Sex** | | | | | | | | |
| Male | 0.83 | 1.25 | 0.40 | 0.53 to 1.28 | - | - | - | - |
| **Ethnicity** | | | | | | | | |
| Non-White | 0.89 | 1.42 | 0.74 | 0.44 to 1.75 | - | - | - | - |
| **Socioeconomic deprivation** | | | | | | | | |
| IMD | 1.03 | 1.06 | 0.57 | 0.93 to 1.15 | - | - | - | - |
| **Diagnoses** | | | | | | | | |
| Diabetes | 0.93 | 1.27 | 0.75 | 0.58 to 1.48 | - | - | - | - |
| Hypertension | 1.12 | 1.26 | 0.63 | 0.71 to 1.77 | - | - | - | - |
| Dementia | 1.31 | 1.48 | 0.49 | 0.59 to 2.79 | - | - | - | - |
| MH diagnosis | 1.02 | 1.36 | 0.94 | 0.55 to 1.85 | - | - | - | - |
| Cancer diagnosis | 1.33 | 1.31 | 0.29 | 0.78 to 2.25 | - | - | - | - |
| HF during admission | 1.6 | 1.29 | 0.07 | 0.96 to 2.63 | 1.53 | 1.28 | 0.09 | 0.94 to 2.49 |
| **Urine ACR** | | | | | | | | |
| ACR > 30 mg/mmol | 0.54 | 1.52 | 0.14 | 0.23 to 1.23 | - | - | - | - |
| ACR 3–30 mg/mmol | 0.64 | 1.38 | 0.18 | 0.34 to 1.22 | - | - | - | - |
| **CKD Stage** | | | | | | | | |
| Advanced CKD  (Stage 4 and 5) | 3.56 | 1.30 | **< 0.0001** | 2.15 to 5.95 | 3.08 | 1.26 | **< 0.0001** | 1.96 to 4.88 |
| **Table Key** | | | | | | | | |
| Reference categories | Age: 1 unit year increase; Sex: Female; Ethnicity: White; Diagnoses: absence of diagnosis; Urine ACR: ACR < 3; CKD stage: stages 1, 2 and 3 combined. SE = standard error | | | | | | | |
| Statistically significant | values in **bold.** | | | | | | | |
